# Supplementary material for: Tunturi virus isolates and metagenome-assembled viral genomes provide insights into the virome of Acidobacteriota in Arctic tundra soils
Source: Microbiome. 2025 Mar 20;13:79. doi: 10.1186/s40168-025-02053-6 (PMC11924767; doi:10.1186/s40168-025-02053-6)
Supplement: Supplementary file 2 — Supplementary Material 1. Figure S1. Similarities between the genomes of Tunturi 1-5 viruses and UViGs retrieved from the IMG/VR database. UViGs IDs are labeled skipping the “IMGVR_UViG_” prefix, for more information about UViGs, see Table S4. Ribbons are colored by % identity (see the key). Minimum and maximum % identities: (A) 64.71 and 100.00, (B) 64.96 and 100.00, (C) 65.11 and 95.83, (D) 64.79 and 90.32, (E) 64.69 and 79.45. Orientation is clockwise for all sequences. Figure S2. Distribution of the Tunturi 1-5 isolates across the 22 Kilpisjärvi meadow and fen metagenomes shown as (A) the percentage of reads mapped to their CDSs and (B) the percentage of detected CDSs per virus. Figure S3. Relative abundance of different taxonomic groups assigned to Kilpisjärvi vOTUs. Figure S4. PCoA of (A) Kilpisjärvi vOTUs (n=1881) and (B) Acidobacteriota-associated vOTUs (n=125) in 22 Kilpisjärvi meadow and fen metagenomes. Convex hulls show actual spread of points. The minimum 50% horizontal coverage was applied. The R2 and p-values were obtained separately for each variable. Figure S5. High-quality (96-100% complete) vOTUs assigned to Acidobacteriota. (A) Genomes with ORFs shown as arrows and colored according to the functional categories (see the color key). (B) Distribution of ORFs according to the functional categories, same color key as in (A). Figure S6. Putative acidobacterial proviruses identified in this study and related vOTUs reported in Emerson et al. 2018 (PMID: 30013236), Candidatus Koribacter versatilis provirus reported in Eichorst et al. 2018 (PMID: 29327410), and NCBI references. ORFs are shown as arrows and similarities between genomes (BLASTn) are in shades of gray (direct) or purple (invert). RC, reverse complement. Figure S7. VConTACT2 viral cluster comprising the isolate Tunturi 3, o12215_NODE_6138 (this study) and three vOTUs reported in Emerson et al. 2018 (PMID: 30013236). Similarities between the genomes (BLASTn) are shown with the shades of gray ( [file 40168_2025_2053_MOESM1_ESM.zip › Supplementary_figures_ESM.docx]

# **Tunturi virus isolates and metagenome-assembled viral genomes provide insights into the virome of *Acidobacteriota* in Arctic tundra soils**

Tatiana Demina^1,2*^, Heli Marttila^1^, Igor S. Pessi^1,2,3^, Minna K. Männistö^4^, Bas E. Dutilh^5,6^, Simon Roux^7^, Jenni Hultman^1,4^

^1^Department of Microbiology, Faculty of Agriculture and Forestry, University of Helsinki, Helsinki, Finland

^2^Helsinki Institute of Sustainability Science (HELSUS), University of Helsinki, Helsinki, Finland

^3^Finnish Environment Institute (Syke), Helsinki, Finland

^4^Natural Resources Institute Finland (Luke), Finland

^5^Institute of Biodiversity, Faculty of Biological Sciences, Cluster of Excellence Balance of the Microverse, Friedrich Schiller University Jena, Jena, Germany

^6^Theoretical Biology and Bioinformatics, Science for Life, Utrecht University, Utrecht, the Netherlands

^7^DOE Joint Genome Institute, Lawrence Berkeley National Laboratory, Berkeley, CA, USA

*corresponding author, tatiana.demina@helsinki.fi

# Supplementary Figures

#
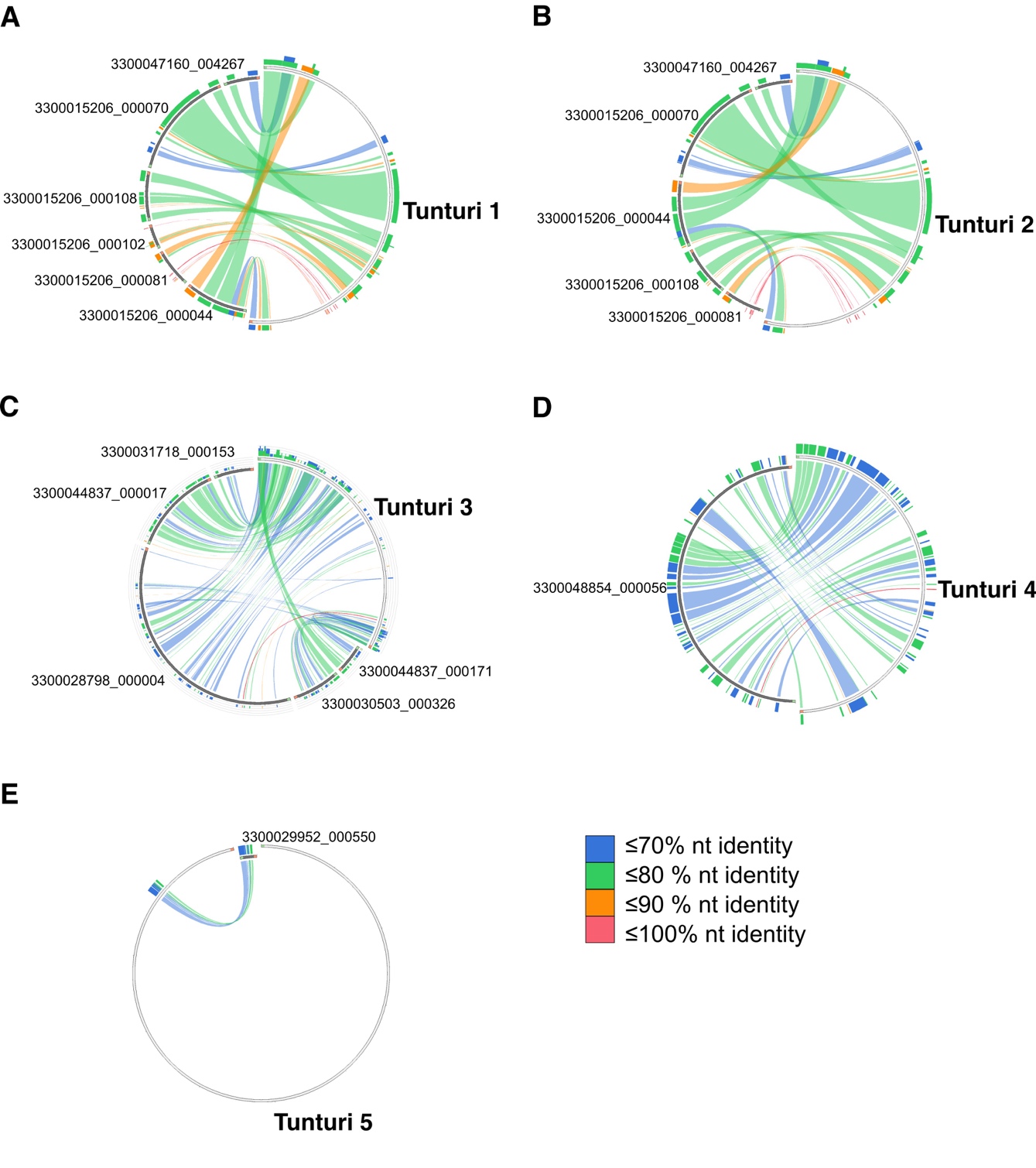
**Figure S1**. Similarities between the genomes of Tunturi 1-5 viruses and UViGs retrieved from the IMG/VR database. UViGs IDs are labeled skipping the “IMGVR_UViG_” prefix, for more information about UViGs, see Table S4. Ribbons are colored by % identity (see the key). Minimum and maximum % identities: (A) 64.71 and 100.00, (B) 64.96 and 100.00, (C) 65.11 and 95.83, (D) 64.79 and 90.32, (E) 64.69 and 79.45. Orientation is clockwise for all sequences.

#
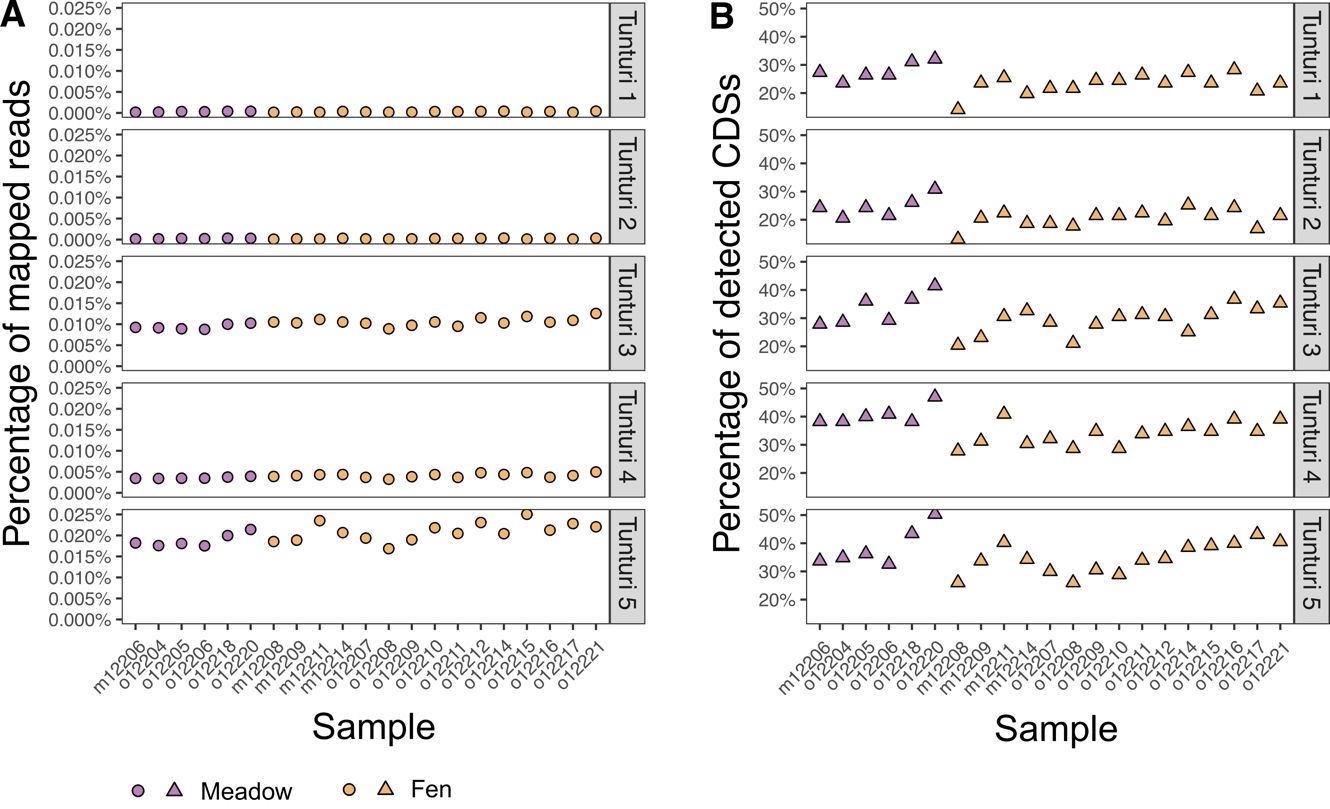


##### **Figure S2**. Distribution of the Tunturi 1-5 isolates across the 22 Kilpisjärvi meadow and fen metagenomes shown as (A) the percentage of reads mapped to their CDSs and (B) the percentage of detected CDSs per virus.


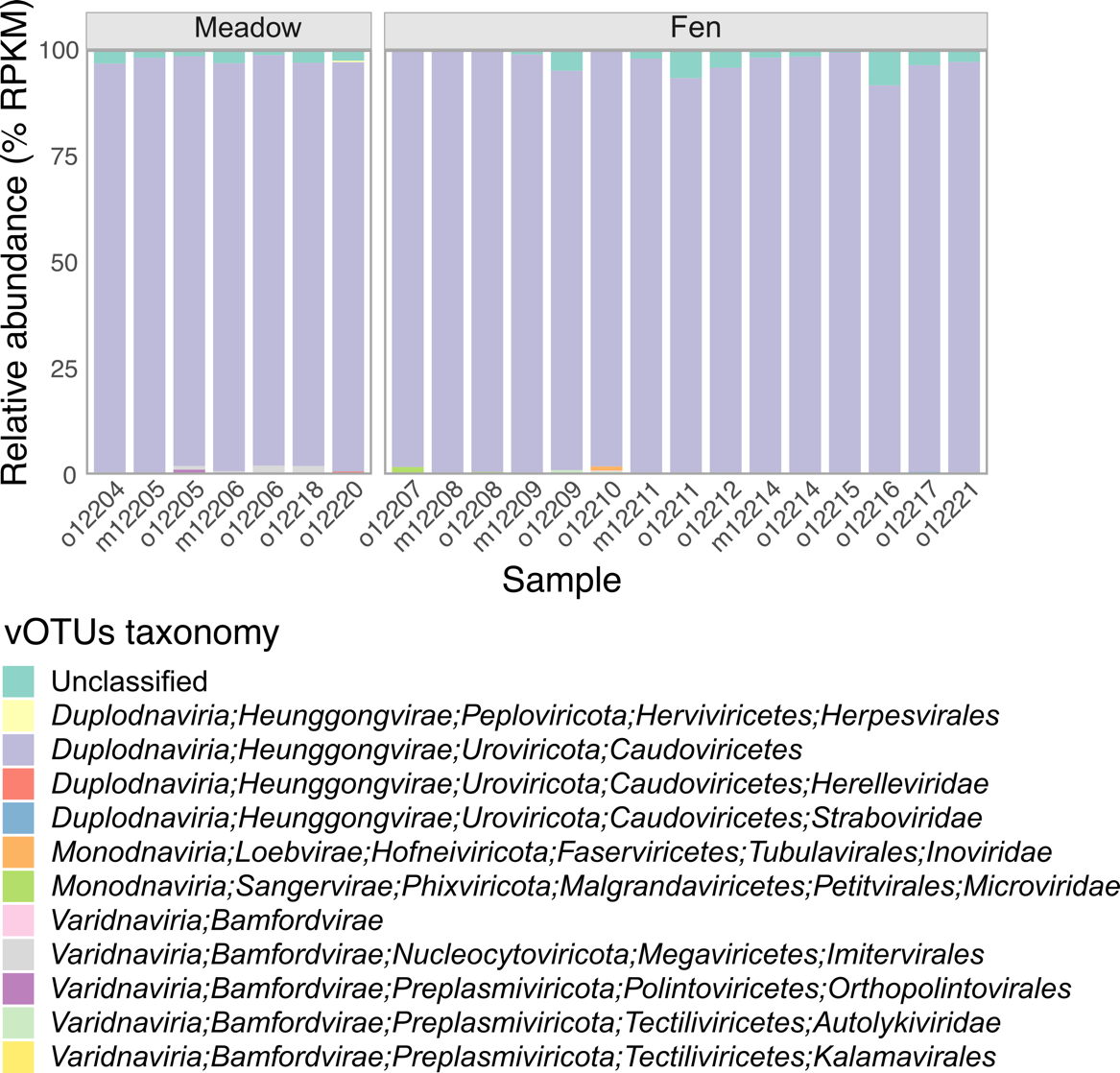


**Figure S3.** Relative abundance of different taxonomic groups assigned to Kilpisjärvi vOTUs.


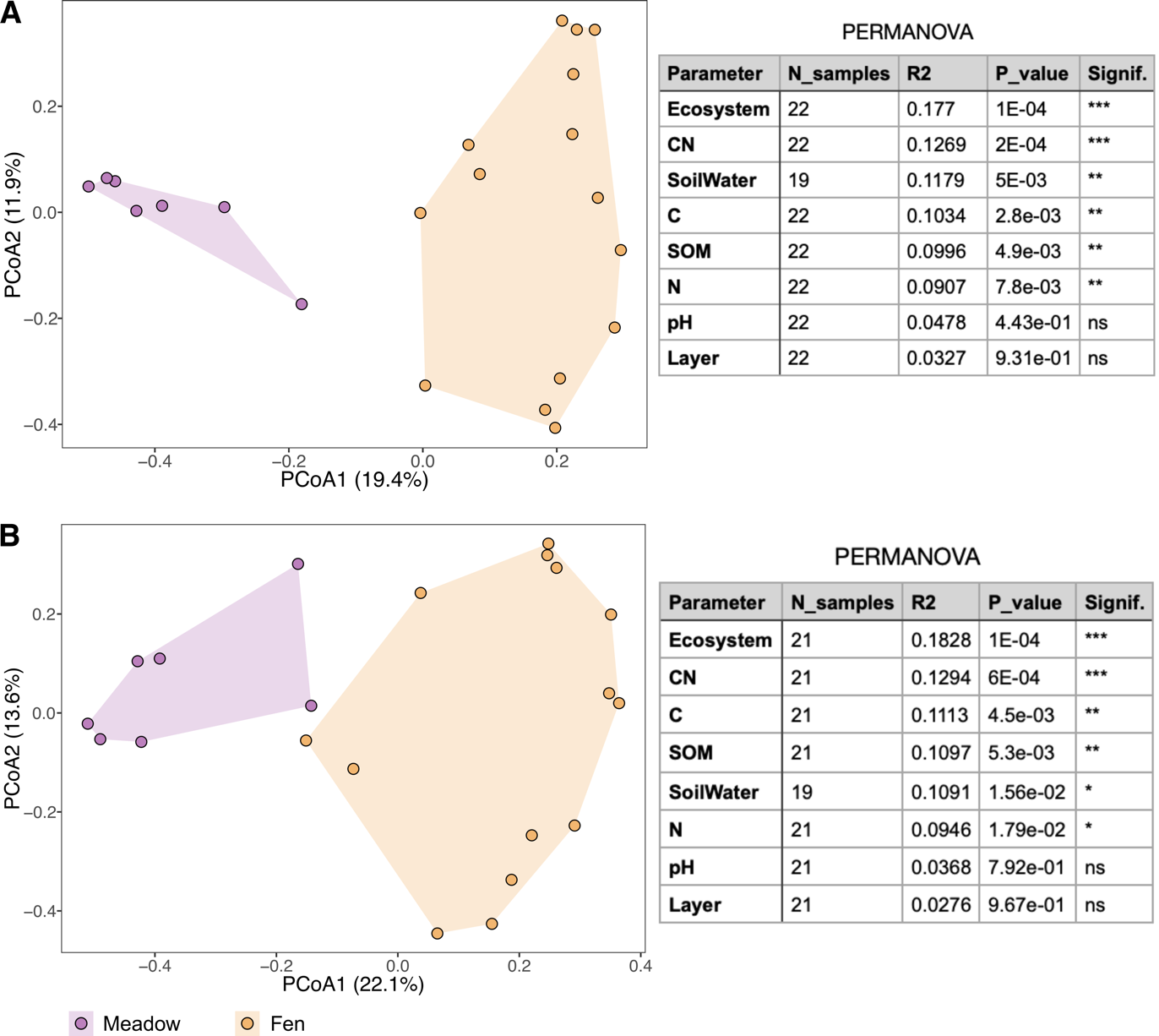


##### **Figure S4.** PCoA of (A) Kilpisjärvi vOTUs (n=1881) and (B) *Acidobacteriota*-associated vOTUs (n=125) in 22 Kilpisjärvi meadow and fen metagenomes. Convex hulls show actual spread of points. The minimum 50% horizontal coverage was applied. The R2 and p-values were obtained separately for each variable.

#####
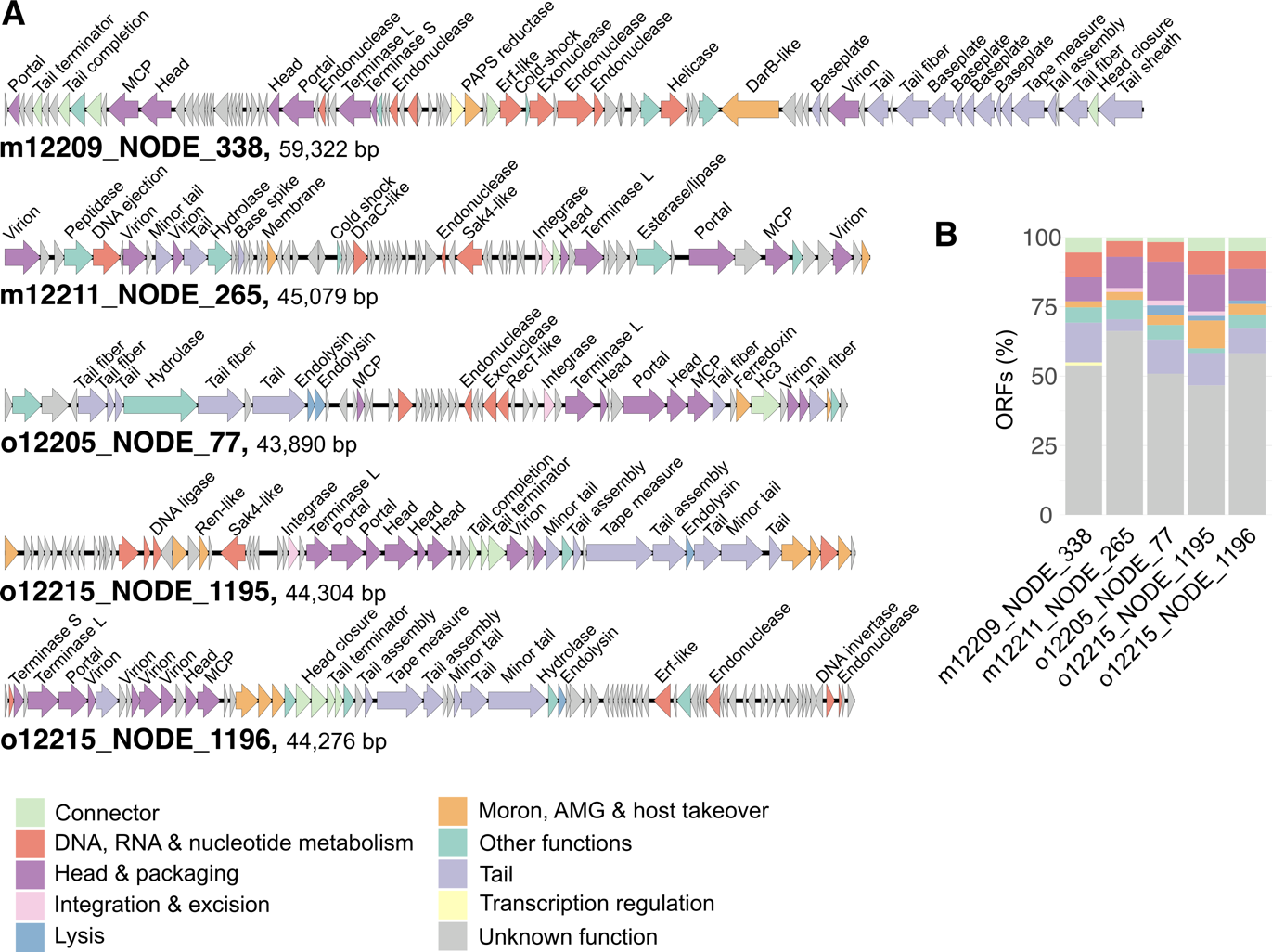


**Figure S5**. High-quality (96-100% complete) vOTUs assigned to *Acidobacteriota*. (A) Genomes with ORFs shown as arrows and colored according to the functional categories (see the color key). (B) Distribution of ORFs according to the functional categories, same color key as in (A).


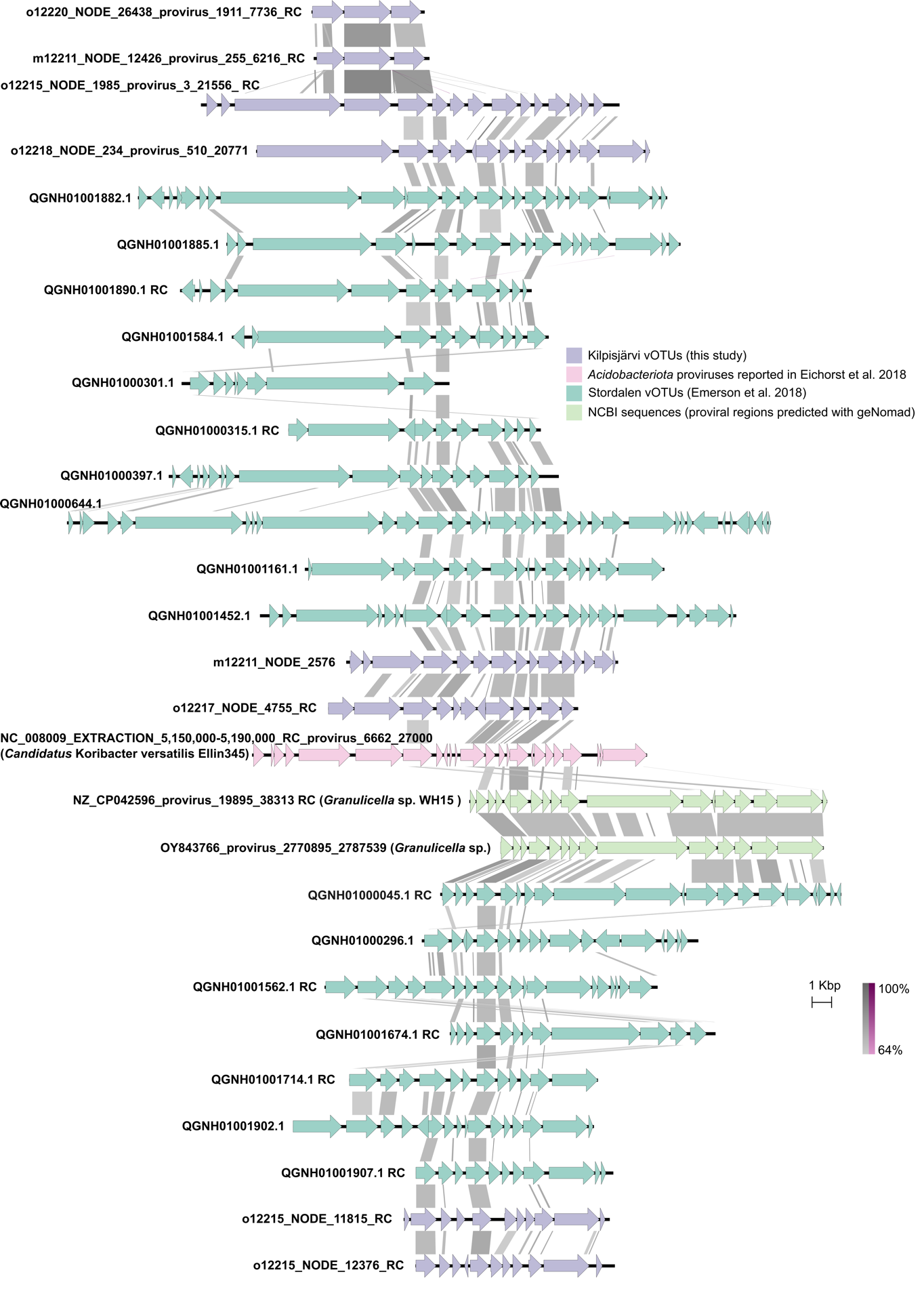


**FigureS6**. Putative acidobacterial proviruses identified in this study and related vOTUs reported in Emerson et al. 2018 (PMID: 30013236), *Candidatus* Koribacter versatilis provirus reported in Eichorst et al. 2018 (PMID: 29327410), and NCBI references. ORFs are shown as arrows and similarities between genomes (BLASTn) are in shades of gray (direct) or purple (invert). RC, reverse complement.


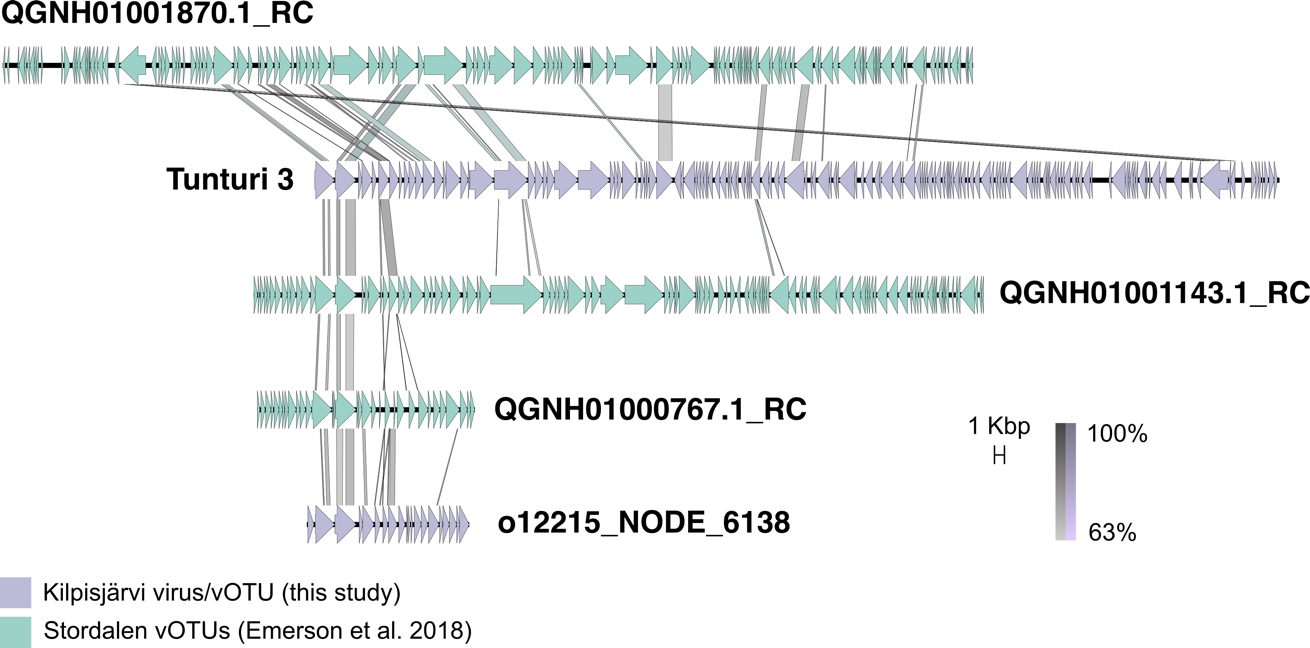


##### **Figure S7**. VConTACT2 viral cluster comprising the isolate Tunturi 3, o12215_NODE_6138 (this study) and three vOTUs reported in Emerson et al. 2018 (PMID: 30013236). Similarities between the genomes (BLASTn) are shown with the shades of gray (direct) or purple (invert).
